# SIRT1 ameliorates oxidative stress induced neural cell death and is down-regulated in Parkinson’s disease

**DOI:** 10.1186/s12868-017-0364-1

**Published:** 2017-06-02

**Authors:** Preeti Singh, Peter S. Hanson, Christopher M. Morris

**Affiliations:** 1 Medical Toxicology Centre, and NIHR Health Protection Research Unit in Chemical and Radiation Threats and Hazards, Newcastle University, Wolfson Building, Claremont Place, Newcastle upon Tyne, NE2 4AA UK; 2 NIHR Biomedical Research Unit in Lewy Body Disorders, and Biomedical Research Centre in Ageing and Chronic Disease, Institute of Neuroscience, Newcastle University, Edwardson Building, Newcastle upon Tyne, NE4 5PJ UK

**Keywords:** SIRT1, Oxidative stress, Cell survival, Alpha-synuclein, Parkinson’s disease

## Abstract

**Background:**

Sirtuins (SIRTs) are NAD^+^ dependent lysine deacetylases which are conserved from bacteria to humans and have been associated with longevity and lifespan extension. SIRT1, the best studied mammalian SIRT is involved in many physiological and pathological processes and changes in SIRT1 have been implicated in neurodegenerative disorders, with SIRT1 having a suggested protective role in Parkinson’s disease. In this study, we determined the effect of SIRT1 on cell survival and α-synuclein aggregate formation in SH-SY5Y cells following oxidative stress.

**Results:**

Over-expression of SIRT1 protected SH-SY5Y cells from toxin induced cell death and the protection conferred by SIRT1 was partially independent of its deacetylase activity, which was associated with the repression of NF-кB and cPARP expression. SIRT1 reduced the formation of α-synuclein aggregates but showed minimal co-localisation with α-synuclein. In post-mortem brain tissue obtained from patients with Parkinson’s disease, Parkinson’s disease with dementia, dementia with Lewy bodies and Alzheimer’s disease, the activity of SIRT1 was observed to be down-regulated.

**Conclusions:**

These findings suggests a negative effect of oxidative stress in neurodegenerative disorders and possibly explain the reduced activity of SIRT1 in neurodegenerative disorders. Our study shows that SIRT1 is a pro-survival protein that is downregulated under cellular stress.

**Electronic supplementary material:**

The online version of this article (doi:10.1186/s12868-017-0364-1) contains supplementary material, which is available to authorized users.

## Background

SIRT1 is a NAD^+^ dependent class III histone deacetylase that shares homology with yeast silent information regulator, SIR2. SIRT1 is primarily a nuclear protein that shuttles to the cytoplasm depending upon the cell type and stress [1]. SIRT1 targets histones and non-histones proteins that are involved in the regulation of several physiological processes including mitochondrial biogenesis, antioxidant defence mechanisms, DNA repair, apoptosis and genomic stability [2]. SIR2 promotes lifespan extension in yeast [3] and brain specific overexpression of its mammalian homologue, SIRT1 has been reported to delay ageing in SIRT1 overexpressing female and male transgenic mice [4]. In support of this protective effect in ageing, SIRT1 is also implicated in life span extension in mice that are either calorie restricted [5] or on standard diet [6]. These studies show that SIRT1 when activated or overexpressed in a tissue-specific manner can slow down ageing and enhance life span.

Parkinson’s disease (PD) is the second most common neurodegenerative disorder after Alzheimer’s disease (AD) and the most common neurodegenerative movement disorder [7, 8]. The characteristic pathological features of the disease are the progressive loss of dopaminergic neurones in the substantia nigra and the accumulation of α-synuclein rich Lewy bodies and Lewy neurites in brain stem, spinal cord and cortical regions [9]. The causes of PD are still unknown, although several environmental and genetic risk factors are suggested as being causative. Ageing is however, the largest single risk factor for the development and progression of neurodegeneration by affecting a number of key cellular processes. At the molecular level, apoptosis, oxidative stress and mitochondrial dysfunction have been shown to promote neurodegeneration. SIRT1 has shown to inhibit apoptosis by regulating p53, reducing oxidative stress by regulating antioxidant defences via FOXO family members, and regulates mitochondrial biogenesis mediated by PGC-1α [10]. SIRT1 also maintains genomic stability by modulating histone acetylation. SIRT1 has been shown to exert neuroprotection against oxidative stress. In models of PD, rotenone and MPP^+^ are known to reduce SIRT1 in primary cultures which may imply that due to reduction of SIRT1, neurones have reduced ability to combat oxidative stress and mitochondrial dysfunction [11]. SIRT1 is neuroprotective by deacetylating and consequently activating PGC-1α and maintains mitochondrial homeostasis by elevating antioxidant defences against MPTP mediated neurotoxicity [12].

Given the possible protective role of SIRT1 in PD, we evaluated the role of SIRT1 and its enzymatic activity in oxidative stress mediated cell death and also characterised the involvement of SIRT1 in PD. The effects of over-expression and deacetylase activity of SIRT1 on cell survival and α-synuclein aggregate formation were determined in diquat or rotenone treated SH-SY5Y cells. Protein expression and activity of SIRT1 was also determined in post-mortem human brain tissue obtained from patients with PD, PD with dementia (PDD), dementia with Lewy bodies (DLB) and AD.

## Methods

### SH-SY5Y cell culture

SH-SY5Y neuroblastoma cells were obtained from the European Collection of Cell Cultures (ECACC, Salisbury, UK) and cultured as described previously [13]. Cells were grown at 37 °C in a humidified atmosphere of 95% air/5% CO_2_.

### SIRT1 overexpression and toxin treatment in SH-SY5Y cells

Wild type SIRT1 (Flag-SIRT1 was a generous gift from Michael Greenberg; Addgene; Plasmid number 1791) and catalytically inactive SIRT1H363Y (Flag-SIRT1 H363Y; Addgene; Plasmid number 1792 from Michael Greenberg) plasmids [14] were obtained from Addgene and sub-cloned in pLenti CMV blast (a gift from Eric Campeau Addgene plasmid number 17486) and empty pLenti CMV blast wild type served as a control [15]. SH-SY5Y cells were seeded in 12 well plates and the cells were transfected with SIRT1 or SIRT1H363Y plasmid (1.5 µg/well) and the control group was transfected with empty pLenti CMV plasmids using PEI (polyethyleneimine; Invitrogen) and plasmids were incubated with cells at 37 °C for 48 h. Stably transfected cells were also studied following selection of lines using 5 µg/ml Blasticidin and amplification of lines. Transfected cells were treated with either diquat (Sigma-Aldrich, UK) dissolved in PBS (phosphate buffered saline; Sigma-Aldrich) or rotenone (Sigma-Aldrich) dissolved in DMSO (dimethyl sulphoxide, Sigma-Aldrich) at a final concentration of 0.2% PBS/DMSO and incubated overnight for 20 h. Cell viability was determined by Alamar Blue reduction assay [13].

### Western blotting

Following Alamar Blue fluorescence, cell lysates were prepared by scraping the viable cells in native lysis buffer (1% 10× tris buffered saline (TBS), 0.27 M Sucrose, 1% Triton X-100, 1× protease inhibitor cocktail). The cell lysates were sonicated for 20 s using a sonic probe and the total protein determined using Bradford Assay (modified from [16]). Twenty micrograms of protein in cell lysates were subjected to electrophoresis and were probed using selected antibodies in a manner as described previously [13] using GAPDH as a housekeeping internal loading control protein.

### Fluorescence immunocytochemistry

SH-SY5Y cells were grown in chamber slides (BD Falcon, UK) and were transfected with SIRT1 plasmids and treated with diquat or rotenone. The cells were washed with PBS and slides incubated with 4% formaldehyde (Sigma-Aldrich) in warm 1× PBS for 15 min and then washed with PBS and stored until use in 10% glycerol (Sigma-Aldrich, UK) at 4 °C. Cells were washed and blocked in 1× PBS/5% goat serum/0.3% Triton X-100 for an hour then incubated overnight at 4 °C with anti-SIRT1 (Santa Cruz Biotechnology) and anti-phospho-α-synuclein (Wako, Saitama, Japan) for α˗synuclein aggregates. Cells were washed with PBS and incubated with secondary antibodies (LifeSciences, Glasgow, UK) for 60 min protected from light. Cells were washed with PBS and counterstained and mounted with ProLong Gold Antifade Mountant with DAPI (Thermo Fisher). Images were acquired using a Zeiss Axioplan 2 microscope (Zeiss, Oberkochen, Germany) with a 40× objective and images captured at 1024 × 1024 pixel resolution for analysis. Images were quantified using ImageJ (NIH, Bethesda, USA). Phospho-α-synuclein aggregate immunoreactivity was determined by using a standardised custom histogram based coloured thresholding technique and then subjected to analyse particles in ImageJ. The parameters recorded were total area and percentage area of staining.

### Post-mortem tissue analysis

Brain samples were obtained from Newcastle Brain Tissue Resource, a Human Tissue Authority licensed tissue bank. Tissue was obtained at post-mortem as soon as possible after death and samples snap frozen and stored at −80 °C. Frozen tissue of the relevant region was identified and protein homogenates from PD, DLB, PDD, AD and control (Table 1) were prepared by homogenising approximately 250 mg of freshly thawed grey matter in 2.5 ml of 0.2 M triethylammonium bicarbonate containing 1× protease inhibitor. After addition of 10 µl of 10% SDS to 500 µl of homogenate, samples were vortexed and then sonicated using a sonic probe for 15 s, followed by sonication on ice in a sonic bath for 40 min. The concentration of protein was determined by Bradford assay. Western blotting was performed as previously [13] using GAPDH as a housekeeping internal loading control protein.

**Table 1:** Details of brain samples used for Western blot and immunohistochemistry

Groups	FCX	TCX	Cb	Pu	Hp	Age at death (years)	Tissue pH	PMD (h)	Gender	
									M	F
Control (N)	11	12	12	12	8	77.5 ± 6.98	6.17 ± 0.34	19.9 ± 6.42	7	5
PD (N)	12	12	12	12	–	77.44 ± 7.03	5.85 ± 0.06	23.44 ± 9.72	8	4
PDD (N)	8	9	12	8	–	75.93 ± 5.38	6.19 ± 0.32	24.69 ± 11.38	9	3
DLB (N)	12	12	12	12	6	77.00 ± 5.35	6.32 ± 0.29	18.0 ± 8.58	9	3
AD (N)	12	12	12	–	9	80.37 ± 5.25	6.19 ± 0.33	19.84 ± 9.30	5	7

Case details of tissue samples used in western blot analysis and immunohistochemistry

*FCX* frontal cortex, *TCX* temporal cortex, *Cb* cerebellum, *Pu* putamen, *Hp* hippocampus, *PMD* post-mortem delay

### Sirtuin activity

Brain protein homogenates were thawed and vortexed and sonicated as previously and samples spun down at 100×*g* at 4 °C for 5 min and the protein concentration of supernatant was determined by Bradford assay. Fluorescent SIRT substrate (p53 379–382), Ac-RHKK(Ac)-AMC was synthesised by Cambridge Research Biolabs, UK. Stock peptide was prepared as a 5 mM solution in diluted SIRT Assay buffer (50 mM Tris–HCl, pH 8.0, containing 137 mM sodium chloride, 2.7 mM potassium chloride, and 1 mM magnesium chloride) and was stored at -70 °C until use. Total SIRT activity was determined by using 30 μg protein in substrate buffer containing 41.6 µM peptide, 1 mM NAD^+^ and 100 nM Trichostatin A (as an Histone Deacetylase inhibitor) and incubated at room temperature for 2 h on a shaker. After 2 h 2.5 μg/ml trypsin in 50 mM nicotinamide (NAM) was added to stop further deacetylation and to cleave the deacetylated product. The fluorescence was recorded for each well after 1 h of incubation of the trypsin-NAM solution in the plate reader on excitation wavelength of 350–360 nm and emission wavelength of 450–460 nm. SIRT1 activity was determined as EX527 (10 μM) inhibitable activity. (Please refer to Additional file 3: Figure S3 for sample and buffer preparation).

### Statistical analyses

Statistical analysis was performed using one-way ANOVA within groups and two-way ANOVA within two groups using SPSS21 (IBM) followed by appropriate post hoc (Bonferroni) non-parametric testing. Error bars represent standard deviation (±SD). p < 0.05 was considered statistically significant. Statistical analysis of Western blotting data was performed in GraphPad Prism using a two samples *t* test assuming unequal variances using protein/GAPDH ratios. Statistical significance was considered as p < 0.05. The results are presented as mean ± SD.

## Results

### SIRT1 reduces toxin induced cell death

**Fig. 1:**
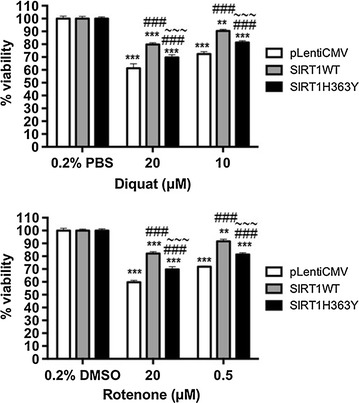
Effect of SIRT1 and its deacetylase activity on cell viability of toxin treated SH-SY5Y cells. SIRT1WT and SIRT1H363Y were over-expressed in SH-SY5Y cells and control cells were transfected with empty pLenti CMV vector following which cells were treated with diquat (20 or 10 μM) or rotenone (20 or 0.5 μM) for 20 h and viability was measured by reduction of Alamar Blue. Data are presented as mean % control ± SD from three independent assays (n = 3). ***p < 0.001 and **p < 0.01 when compared to 0.2% vehicle (PBS/DMSO), one-way ANOVA (Bonferroni corrected), ^###^p < 0.001 and ^##^p < 0.01 when compared to empty vector treatment and ^~~~^p < 0.001 when compared to SIRT1WT, two-way ANOVA (Bonferroni corrected)

Exposure to environmental factors, such as rotenone and diquat has been shown to lead to oxidative damage in dopaminergic neurones leading to parkinsonian symptoms in animal and cellular models [17]. In diquat treated cells, SIRT1WT transfected cells (see Additional file 1: Figure S1) showed increased rates of cell survival compared to control cells (20 µM or 10 µM diquat: p < 0.001) and SIRT1H363Y cells (20 µM diquat: p < 0.01; 10 µM diquat: p < 0.001). Interestingly, increased cell viability was observed in SIRT1H363Y transfected cell compared to control cells (20 µM or 10 µM diquat: p < 0.001) (Fig. 1). In cells treated with rotenone, SIRT1WT and SIRT1H363Y overexpression enhanced cell viability (rotenone 20 μM or 0.5 μM rotenone—p < 0.001) compared to control cells where SIRT1WT overexpression was more potent in combating oxidative stress (Fig. 1). Similar findings to transiently transfected cells were found in stably transfected cells (data not shown).

### SIRT1 protects SH-SY5Y cells by reducing expression of NF-кB and cleaved PARP-1

**Fig. 2:**
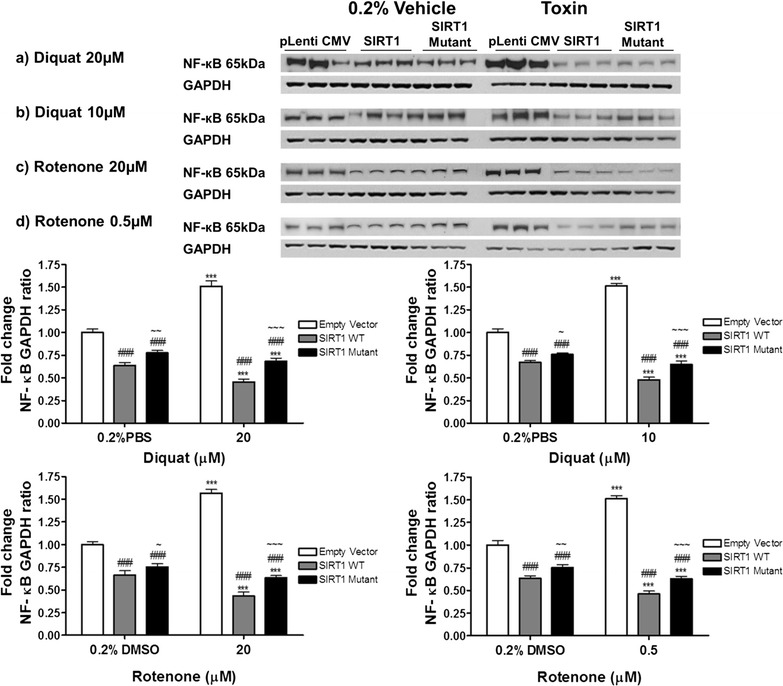
Expression of NF-κB in toxin treated SH-SY5Y. SIRT1WT and SIRT1H363Y were over-expressed in SH-SY5Y cells and control cells were transfected with empty vector following which cells were treated with diquat (**a** 20 or **b** 10 μM) or rotenone (**c** 20 or **d** 0.5 μM) for 20 h. Cells were harvested and the samples were probed for NF-κB. Data are presented as fold-untreated (+SD) from three independent assays (n = 3) with comparison to GAPDH as a housekeeping control protein. ***p < 0.001 when compared to 0.2% PBS, one-way ANOVA (Bonferroni corrected), ###p < 0.001 when compared to empty vector treatment, ^~~~^p < 0.001, ^~~^p < 0.01 and ^~^p < 0.05 when compared to SIRT1WT cells, two-way ANOVA (Bonferroni corrected). Images are representative blot of NF-κB and GAPDH

**Fig. 3:**
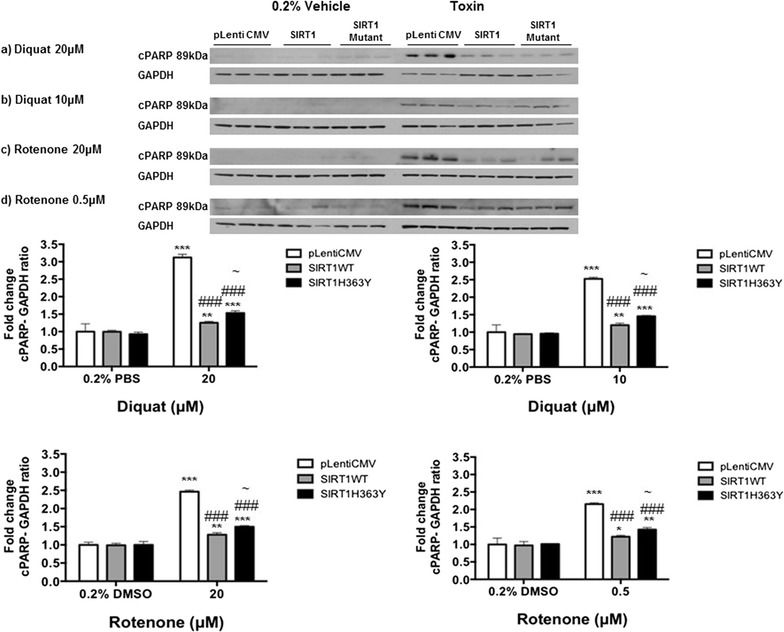
Expression of cleaved PARP-1 in toxin treated SH-SY5Y. SIRT1WT and SIRT1H363Y were over-expressed in SH-SY5Y cells and control cells were transfected with empty vector following which cells were treated with diquat (**a** 20 or **b** 10 μM) or rotenone (**c** 20 or **d** 0.5 μM) for 24 h. Cells were harvested and the samples were probed for cPARP-1. Data are presented as fold-untreated (+SD) from three independent assays (n = 3) with comparison to GAPDH as a housekeeping control protein. ***p < 0.001 and **p < 0.01 when compared to 0.2% PBS, one-way ANOVA (Bonferroni corrected), ^###^p < 0.001 when compared to empty vector treatment, ^~^p < 0.05 when compared to SIRT1WT cells, two-way ANOVA (Bonferroni corrected). Images are representative blot of cPARP1 and GAPDH

Overexpression of SIRT1 in diquat or rotenone treated SH-SY5Y cells, rescued cells from oxidative stress. To test the possible mechanism behind this protection, cells were probed for NF-κB. The levels of NF-κB were reduced by 32–35% in SIRT1WT (p < 0.001) and by 23–24% in SIRT1H363Y (p < 0.001) cells treated with 0.2% PBS compared with pLenti CMV transfected cells (Fig. 2). In diquat treated cells, the levels of NF-κB were reduced by 50–55% in SIRT1WT (20 µM or 10 µM diquat, p < 0.001) cells and by 35–40% in SIRT1H363Y (20 µM or 10 µM diquat, p < 0.001) when compared to 0.2% PBS treated control cells. On the other hand, in pLenti CMV transfected cells, diquat treatment enhanced the level of NF-κB by ~50% (20 µM or 10 µM diquat, p < 0.001) compared to 0.2% PBS treatment (Fig. 2). In rotenone treated cells, the levels of NF-κB in 0.2% DMSO treated cells were reduced by ~34 and ~25% in SIRT1WT (p < 0.001) and SIRT1H363Y (p < 0.001) transfected cells, respectively, compared to pLenti CMV transfected cells (Fig. 1). Following rotenone treatment, the levels of NF-κB were reduced by approximately 55% in SIRT1WT cells (20 or 0.5 µM rotenone, p < 0.001) and by approximately 35% in SIRT1H363Y (20 or 0.5 µM rotenone, p < 0.001), whilst in pLenti CMV transfected cells the levels were elevated by 55–60% in rotenone treated cells (20 or 0.5 µM rotenone, p < 0.001; Fig. 1). Following toxin treatments (diquat: 20 or 10 µM and rotenone: 20 or 0.5 µM) in all cells the levels of SIRT1 were reduced by 45–50% compared to 0.2% vehicle treatment (p < 0.01, Additional file 1: Figure S1). We also analysed the expression of active cleaved Poly (ADP-ribose) polymerase (PARP-1) under oxidative stress and observed that SIRT1 overexpression reduced the protein level of cPARP-1 (diquat: 20 or 10 µM and rotenone: 20 or 0.5 µM by 50–60%; p < 0.001) and similar result was also observed in SIRT1H363Y transfected cells, (diquat: 20 or 10 µM and rotenone: 20 or 0.5 µM by 40–45%; p < 0.001), compared to pLenti CMV transfected cells (Fig. 3). Western blot analysis failed to detect the levels of cleaved caspase 3, a finding that has been observed previously [13].

### SIRT1 shows minimal co-localisation with α-synuclein but reduces aggregate formation

**Fig. 4:**
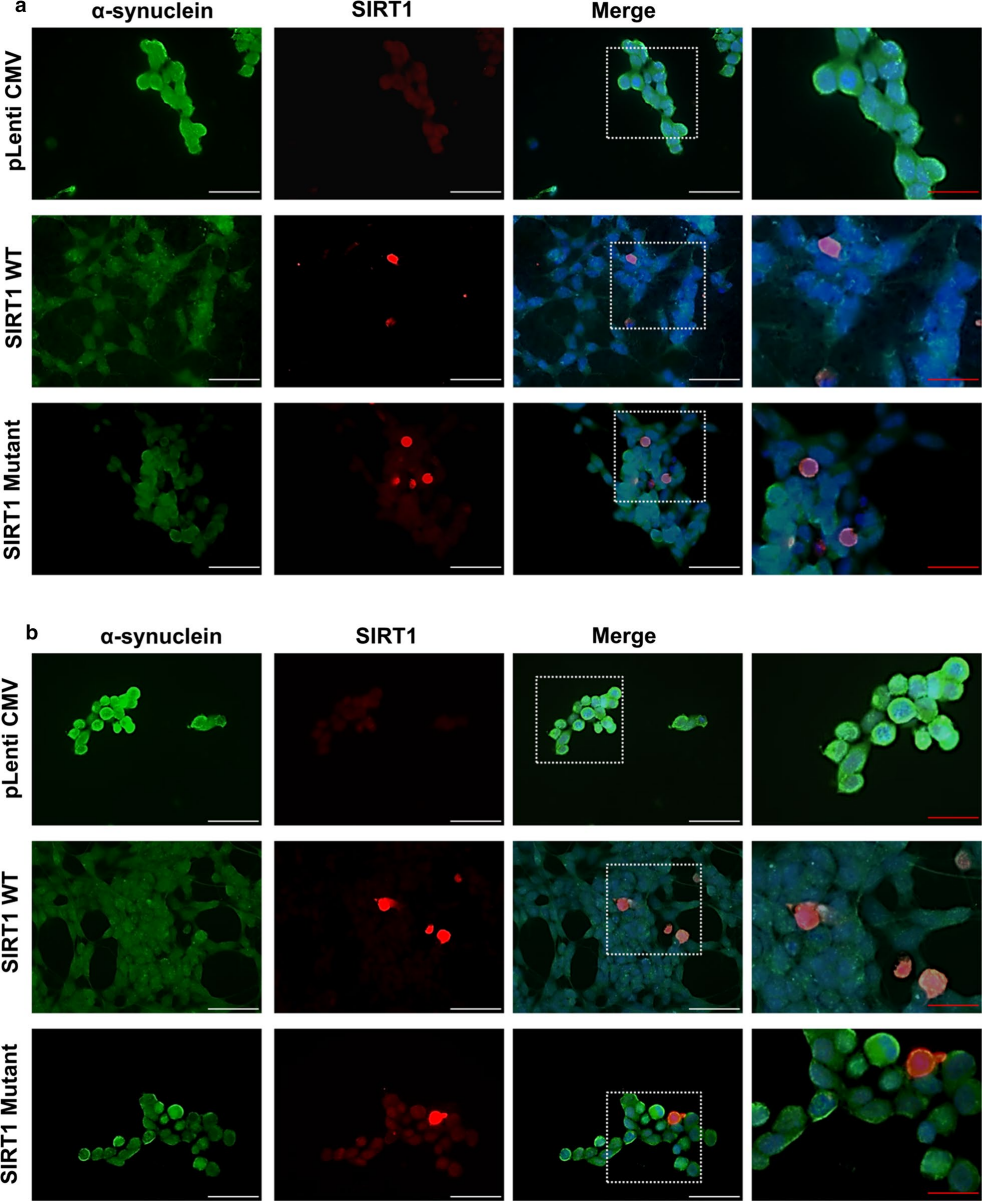
Localisation of SIRT1 and phospho-α-synuclein in toxin treated SH-SY5Y cells. Cellular distribution of SIRT1 and phospho-α-synuclein was determined using fluorescent immunocytochemistry. Images show α-synuclein immunostaining, SIRT1 immunostaining and all staining merged including DAPI in **a** 20 μM diquat and **b** 20 μM rotenone treated cells. *Scale bars*—*white scale bar* 50 μM and *red scale bar* 20 μM; magnification: ×40

SIRT1 is generally localised to the nucleus and depending upon environmental cues and cell types, and can translocate to the cytoplasm [1] and also to mitochondria [18]. Nuclear SIRT1 targets the proteins involved in DNA repair, antioxidant defence, genomic stability and apoptosis [19], whereas localisation of SIRT1 in the cytoplasm augments cells sensitivity to apoptosis [20]. In diquat or rotenone treated cells, SIRT1 was predominantly localised to the nucleus with lower levels of protein observed in the cytoplasm in pLenti CMV, SIRT1WT or SIRT1H363Y transfected SH-SY5Y cells (Fig. 4). Nuclear localisation of SIRT1 correlates with its pro-survival activity under oxidative stress. In the nucleus, SIRT1 targets FOXO family members and promotes the expression of proteins involved in antioxidant defence mechanisms and simultaneously inhibits the expression of targets involved in promotion of apoptosis [14] and also enhances DNA repair [21].

**Fig. 5:**
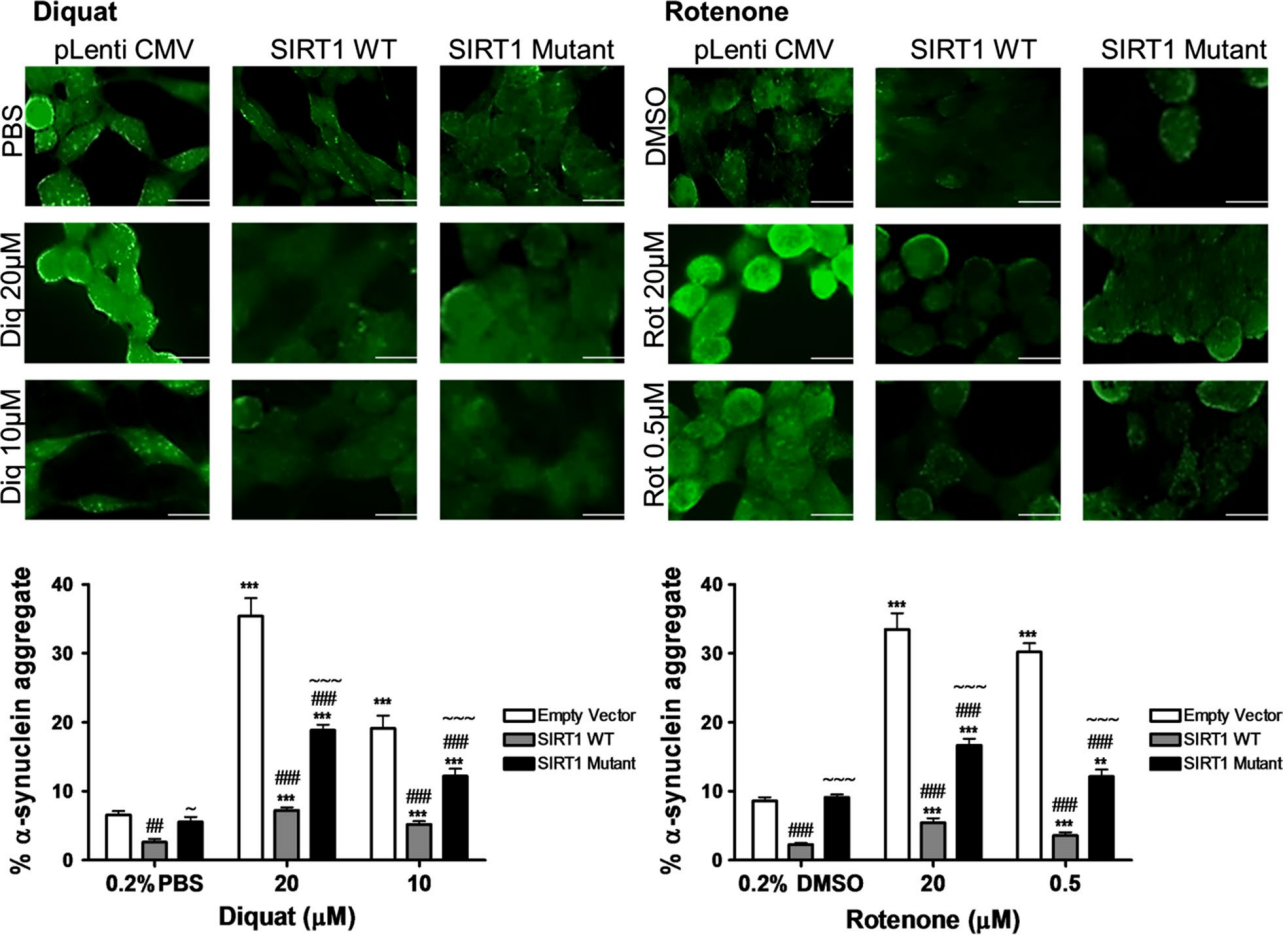
Phospho-α-synuclein aggregate formation in toxin treated SH-SY5Y cells. SIRT1WT and SIRT1H363Y overexpressing SH-SY5Y cells were treated with toxin (20 or 10 μM diquat or 20 or 0.5 μM rotenone) and 0.2% PBS or DMSO; cells transfected with empty vector were used as a control. Cells were immunostained with phospho-α-synuclein. Images were captured through GFP filter under ×63 magnification. The captured images represent phospho-α-synuclein staining and the *bar graphs* represent the aggregate quantification in diquat or rotenone treated cells. *Each bar* represents % phospho-α-synuclein aggregates (±SD) from three independent assays (n = 3). ***p < 0.001, **p < 0.01 and *p < 0.05 when compared to 0.2% vehicle, one-way ANOVA (Bonferroni corrected), ^###^p < 0.001 and ^##^p < 0.01 when compared to control cells, ^~~~^p < 0.001 and ^~^p < 0.05 when compared to SIRT1WT overexpressing cells, two-way ANOVA (Bonferroni corrected). *Scale bar* 20 μM

Under basal conditions, we observed small punctate cytoplasmic accumulations of phosphor-α-synuclein staining which was reduced following SIRT1WT treatment (p < 0.01, Fig. 5) but not with catalytically inactive SIRT1H363Y transfected cells. On treatment with diquat or rotenone, a significant reduction in the number of phospho-α-synuclein aggregates was observed in both SIRT1WT and SIRT1H363Y transfected cells compared to pLenti CMV transfected cells (p < 0.001; Fig. 5). SIRT1 showed minimal co-localisation with phospho-α-synuclein hence its effect on aggregate formation could possibly be through a positive action on ROS scavenging. Compared to SIRT1WT transfected cells, phospho-α-synuclein aggregation was higher in SIRT1H363Y transfected cells (p < 0.001; Fig. 5).

### SIRT1 protein in neurodegenerative disorders

We determined SIRT1 protein and enzyme activity in post mortem human brain tissue in PD and other neurodegenerative disorders. Two isoforms of SIRT1 protein are known (UniProt Q96EB6 (SIRT1_HUMAN), SIRT1FL; predicted molecular weight 80 kDa) however the observed molecular weight on Western blot is ~120 kDa possibly due to post translational modifications [22] along with a shorter form, isoform 2, of approximately 75 kDa. The 120 kDa SIRT1 isoform was observed only in frontal cortex samples and SIRT1 isoform 2 was observed only in the temporal cortex samples.

**Fig. 6:**
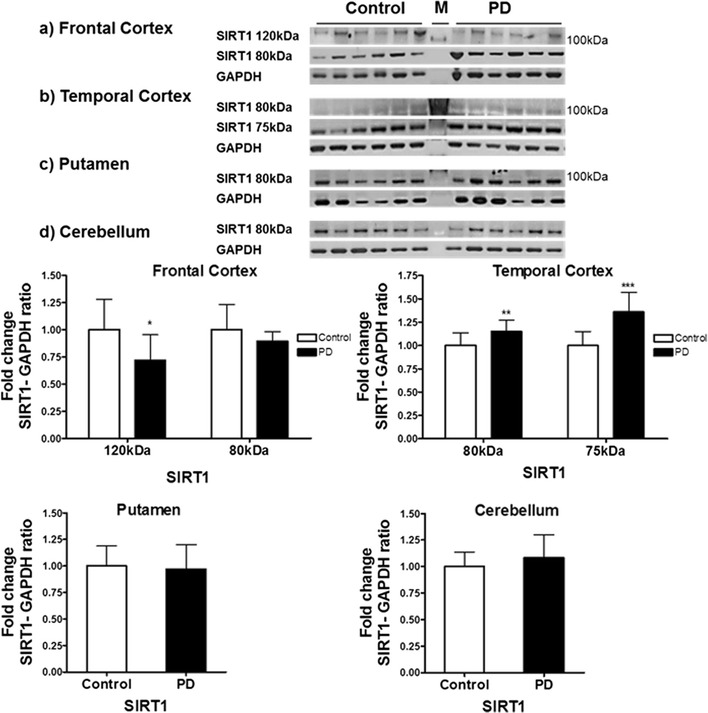
Expression of SIRT1 protein in post mortem brain in Parkinson’s Disease. The levels of SIRT1 were determined in different regions of Parkinson’s disease (PD) patients and compared to a control-cohort. SIRT1 band intensity was normalised with GAPDH as a housekeeping protein. Data are presented as fold change (±SD) with respect to control from three independent replicates of SIRT1/GAPDH. ***p < 0.001, **p < 0.01 and *p < 0.05 (*t* test). Images are representative blots of SIRT1 and GAPDH. M denotes molecular weight marker lane

In the frontal cortex samples of PD, the levels of 120 kDa SIRT1 were reduced by 28% compared to controls (p < 0.05) whereas no significant difference was observed in the levels of SIRT1FL (80 kDa). In the temporal cortex samples from PD cases, the levels of 80 kDa SIRT1FL were elevated by 15% (p < 0.01) and 75 kDa isoform 2 were elevated by 36% (p < 0.001) compared to controls. In the putamen or cerebellum of PD cases, no significant differences were observed in the levels of 80 kDa SIRT1FL isoform when compared to control (Fig. 6).

**Fig. 7:**
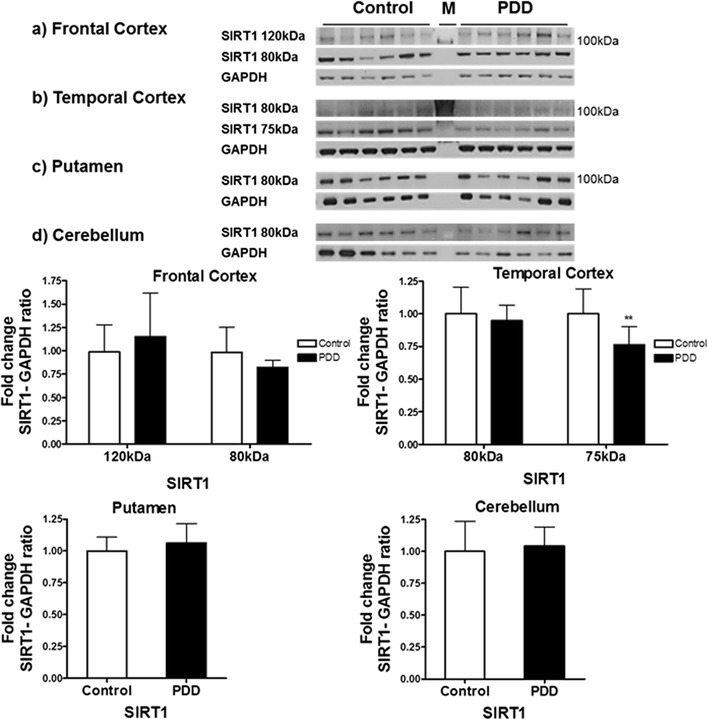
Expression of SIRT1 protein in post mortem brain of Parkinson’s disease with dementia. The levels of SIRT1 were determined in different regions of Parkinson’s disease with dementia patients and compared to a control cohort. SIRT1 band intensity was normalised with GAPDH as a housekeeping protein. Data are presented as fold change (±SD) with respect to control from three independent replicates. **p < 0.01 (*t* test). Images are representative blots of SIRT1 and GAPDH. M denotes molecular weight marker lane

In the frontal cortex of PDD, no significant differences were observed in the levels of 80 kDa SIRT1FL and 120kDaSIRT1 compared to control. In the temporal cortex of PDD, no significant difference was observed in the level of SIRT1FL but the level of the 75 kDa isoform 2 was reduced by 26% compared to control (p < 0.01). In the putamen or cerebellum of PDD cases, no significant differences were observed in the levels of SIRT1 when compared to control (Fig. 7).

In the frontal cortex of DLB, no changes were observed in the levels of 120 kDaSIRT1 whereas, SIRT1FL was up regulated by 22% (p < 0.05). In the temporal cortex of DLB, SIRT1FL was up regulated by 38% (p < 0.001) whilst, no changes were observed in the levels of isoform 2. In the putamen or hippocampus of DLB cases, no significant difference was observed in the levels of SIRT1 when compared to control. The expression of SIRT1 was up regulated by 21% (p < 0.05) in the cerebellum of DLB cases compared to control (Additional file 2: Figure S2).

In the frontal cortex of AD, the levels of 120 kDa SIRT1 and 80 kDa SIRT1FL were reduced by 30% (p < 0.01) and 26% (p < 0.01), respectively, when compared to control. In the temporal cortex of AD, the levels of 80kDa SIRT1FL were reduced by 25% (p < 0.01), whereas, no significant change was observed in the level of isoform 2. In the hippocampal and cerebellar samples of SD, the levels of SIRT1 were reduced by 36% (p < 0.01) and 30% (p < 0.001), respectively, compared to control (Additional file 3: Figure S3).

### SIRT1 activity in neurodegenerative disorders

**Fig. 8:**
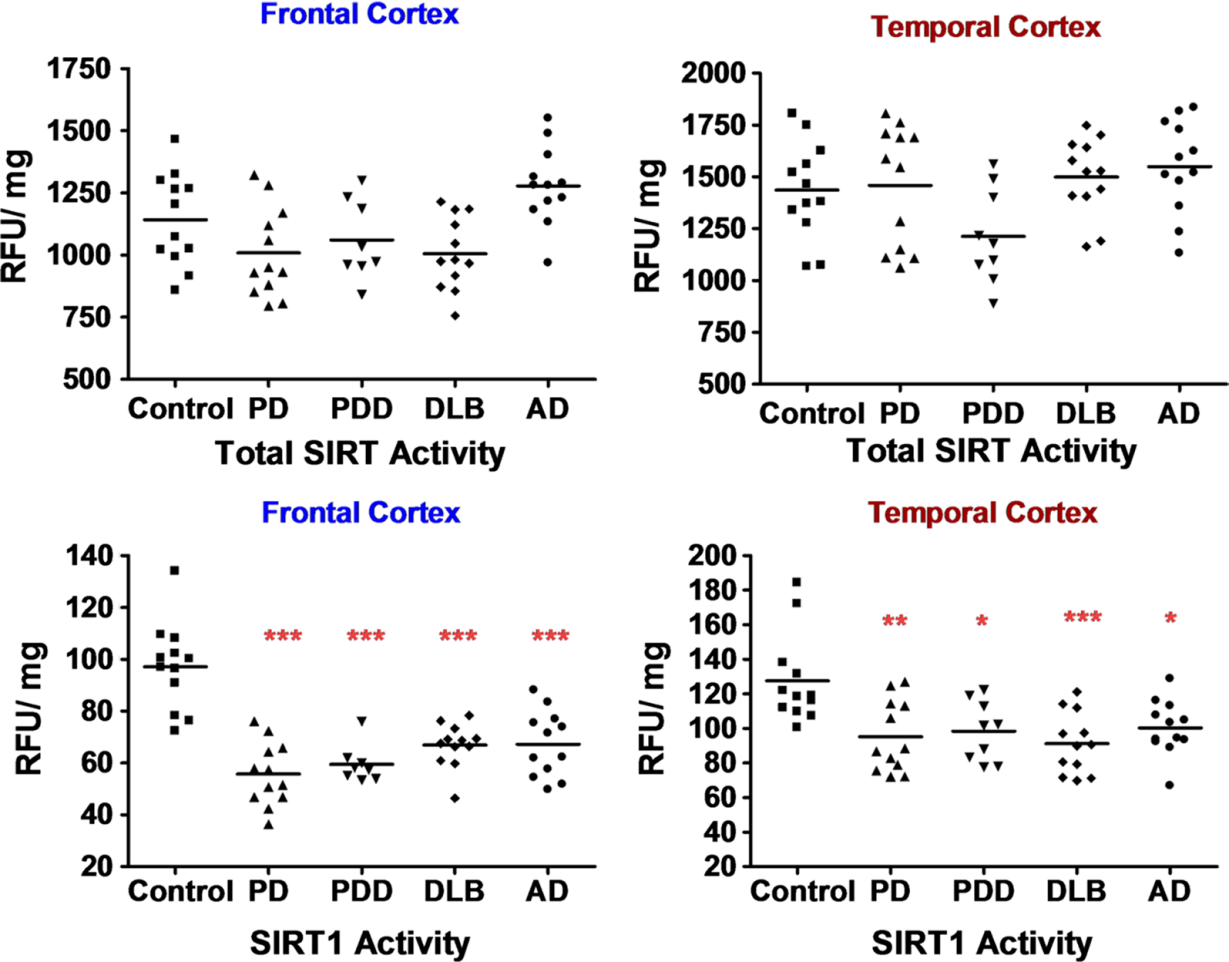
Total sirtuin activity and specific SIRT1 activities in frontal and temporal cortex in neurodegeneration. Total SIRT and SIRT1 activities were measured using a fluorometric enzymatic activity assay with specific SIRT1 inhibition in the frontal and temporal cortices of PD, PDD, DLB and AD patients and were compared to cohort-control group. ***p < 0.001, **p < 0.01 and *p < 0.05 when compared to control, one-way ANOVA (Bonferroni corrected)

In the frontal cortex, measurement of total SIRT activity did not show any significant change between the disease groups and control (p > 0.05); however, compared to AD, the total SIRT activity was reduced in PD and DLB by about 20% (p < 0.01), though PDD did not show any significant difference. Specific SIRT1 activity was down regulated in PD (43%; p < 0.001), PDD (39%; p < 0.001), DLB (32%; p < 0.001) and AD (31%; p < 0.001) compared to controls (F 20.457, p < 0.001). In the temporal cortex, there was no significant difference in total SIRT activity between the disease groups and control (p > 0.05), however, compared to AD there was a significant reduction of 33% in total SIRT activity in PDD (p < 0.05), though other groups did not show any significant change. SIRT1 activity was reduced in PD (25%; p < 0.01), PDD (23%; p < 0.05), DLB (30%; p < 0.001) and AD (22%; p < 0.05) compared to controls (F: 6.265, p < 0.001) whereas no significant difference was seen among the disease groups (Fig. 8).

## Discussion

Oxidative stress is an imbalance between the production of free radicals and antioxidant defence that leads to DNA, protein and lipid damage that may eventually lead to cell death. Oxidative stress has been suggested to play a role in the development and progression of neurodegenerative disorders [23]. SIRT1, a NAD^+^ dependent deacetylase, is a redox sensor and has been shown to regulate key cellular processes including antioxidant defence, DNA repair and genomic stability [2]. In this context, we determined the role of SIRT1 in cell survival under oxidative stress. The results of our study showed increased cell survival in SIRT1WT and SIRT1H363Y transfected cells compared to control cells, nonetheless, SIRT1WT showed the highest rate of cell survival. In SN4741 DA neurones, Mudo et al. [12] reported the protective effect of SIRT1 following MPTP treatment cells which was mediated through activation of PGC-1α leading to enhanced expression of the mitochondrial antioxidants manganese superoxide dismutase and Thioredoxin-2. In a recent study it was shown that SIRT1 alleviated rotenone induced cell death in SH-SY5Y cells by regulating expression of histone H3 and p53 [24]. The present results are in line with these findings and establishes the pro-survival role of SIRT1 against oxidative stress induced by diquat or rotenone in SH-SY5Y cells.

The findings of our study also show that the protection conferred by SIRT1 was partly independent of its deacetylase activity as the enzymatic inactive SIRT1 H363Y mutant also protected the cells from diquat or rotenone induced cell death. Similar to the findings of this study, Pfister et al., showed that SIRT1WT and mutant forms lacking deacetylase activity (H363Y and H355A) protected cerebellar granule neurones from low-potassium induced toxicity, though in their study the extent of protection by mutant forms was similar to SIRT1WT [25]. The difference between their and our study could be attributed to the type of cell used and also the trigger causing cellular stress but does indicate that effects of SIRT1 can be independent of deacetylase activity.

The protection conferred by SIRT1 appears to be mediated at least in part through suppression of NF-кB expression, similar to the effect of SIRT1 on NF-кB observed by Ghosh et al., who reported that SIRT1 and SIRT1H363Y repressed NF-кB activity [26]. NF-кB transcription factors regulate inflammation and immunity processes [27] and also play an important role in cell growth and development, and apoptosis [28]. ROS in cells are known to activate NF-кB leading to transactivation of targets involved in protection against ROS [29] and under high oxidative stress, activation of NF-кB enhances cell death [30]. The results from our study show that SIRT1 protects cells from oxidative stress in part by repressing the expression of NF-кB suggesting that possibly NF-кB enhances cell death on treatment with diquat or rotenone.

PARP-1 (116 kDa) is a nuclear protein involved in detection and repair of DNA damage and is also activated during apoptosis and necrosis [13, 31] and SIRT1 conferred protection to cells by reducing the levels of cleaved PARP-1 (89 kDa) following diquat or rotenone exposure. Rajamohan et al. [32] showed that SIRT1 deacetylates PARP-1 under stress resulting in reduction of PARP-1 mediated cell death. They also observed that SIRT1 negatively regulates the PARP-1 gene promoter and controls the PARP-1 expression at a transcriptional level. The results from our study are in accord with these findings with reduced levels of cleaved PARP-1 protein in both SIRT1 WT and SIRT1H363Y cells possibly via a mechanism independent of the deacetylase activity of SIRT1 through transcriptional changes.

Lewy bodies (LBs) composed of abnormally folded and aggregated α-synuclein proteins, are a characteristic feature of PD and other Lewy bodies disorders [33]. α-synuclein is a small pre-synaptic protein that undergoes oligomerisation and aggregation resulting in fibrillation and formation of LBs. Several factors trigger oligomerisation of α-synuclein including oxidative stress and in this study, we observed a low level of basal phosphor-α-synuclein accumulation which was reduced slightly by SIRT1 overexpression. We also observed that under conditions of oxidative stress, over-expression of SIRT1WT reduced the formation of phospho-α-synuclein aggregates and SIRT1H363Y showed a similar effect when compared to control cells. Interestingly, SIRT1 was not seen to co-localise with phospho-α-synuclein suggesting that the effect of SIRT1 on the aggregate formation is indirect, independent of deacetylase activity, possibly through elevation of cellular anti-oxidant defence mechanisms.

Neurodegenerative disorders typically present pathologically with loss of neurones in the CNS. The causes of neurodegenerative disorders are still under investigation, although environmental and known genetic risk factors are suggested to initiate these disorders, with oxidative stress being associated with initiation and progression of neurodegenerative disorders [34]. SIRT1 has been shown to modulate and repress the damage caused by oxidative stress. In this study, no major alterations were observed in the levels of SIRT1 protein in PD or PDD, whilst the levels of SIRT1 showed a slight non-significant elevation in DLB. However, in AD, the levels of SIRT1 protein were reduced in all brain regions. Pallas et al., analysed the expression of SIRT1 in PD (n = 3), DLB (n = 4) in the frontal cortex compared to controls (n = 4) and observed no changes between the groups [11]. In the current study no major difference was seen in expression of SIRT1 protein in PD but slightly elevated levels of the protein were observed in DLB. The inconsistencies between the previous and current studies could possibly be explained by group sizes with in the current study, the number of cases being larger. A significant reduction in the level of SIRT1 protein was observed in AD and this is most likely explained by the greater degree of cortical atrophy seen in AD and the loss of neuronal or synaptic SIRT1 [35]. To further assess the role of SIRT1, activity was measured and compared to control, with SIRT1 activity reduced in all disease groups although no significant difference was seen between disease groups. The lower SIRT1 activity in all disease groups correlates with possible higher oxidative stress, synaptic and cell loss, and neuroinflammation in neurodegeneration, and also with current findings of reduced SIRT1 levels in SH-SY5Y cells during oxidative stress (see Additional file 1: Figure S1). The differences in enzyme activity and protein expression may be related to the different isoforms found in brain tissue having different enzymatic effects with some isoforms being more catalytically active than others. Studies have reported that oxidative stress and inflammation are upregulated in neurodegenerative disorders [36, 37]. SIRT1 has been shown to alleviate the damage induced by oxidative stress and neuroinflammation. Thus, down regulation of SIRT1 protein and activity in disease groups could be associated with generalised neurodegeneration observed in these disorders and the associated synaptic and neuronal loss induced by chronic oxidative stress and neuroinflammation.

## Conclusion

In this study, we have shown that over-expression of SIRT1 protected SH-SY5Y cells from diquat or rotenone by down-regulating NF-кB and cPARP-1 and reducing phospho-α-synuclein aggregates. The observed protection exerted by SIRT1 is not entirely dependent on its deacetylase activity as the enzymatically inactive mutant of SIRT1 also conferred a degree of protection to cells. In post-mortem human brain tissue, expression of SIRT1 protein did not differ markedly in Lewy body disorders whilst reductions in SIRT1 expression was observed in AD, though the enzymatic activity was down-regulated in all groups compared to controls. Based upon these findings it can be concluded that SIRT1 is a pro-survival protein which is down-regulated under oxidative stress.

## Additional files

**Additional file 1: Figure S1.** Expression of SIRT1 in toxin treated SH-SY5Y cells. SIRT1WT and SIRT1H363Y were over-expressed in SH-SY5Y cells and control cells were transfected with empty vector following which cells were treated with diquat (20 or 10 μM) or rotenone (20 or 0.5 μM) for 20 h. Cells were harvested and the samples were probed for SIRT1. Data are presented as fold- untreated (+SD) from three independent assays (n = 3) with comparison to GAPDH as a housekeeping control protein. ***p < 0.001 when compared to 0.2% PBS, one-way ANOVA (Bonferroni corrected), ###p < 0.001 when compared to empty vector treatment, two-way ANOVA (Bonferroni corrected). Images are representative blot of SIRT1 and GAPDH.

**Additional file 2: Figure S2.** Expression of SIRT1 in different regions of DLB and control brain tissue. The levels of SIRT1 were determined in different regions of DLB patients and were compared to a control-cohort. SIRT1 band intensity was normalised with GAPDH. Data are presented as fold change (±SD) with respect to control from three independent replicates with comparison to GAPDH as a housekeeping control protein. **p < 0.01 and *p < 0.05 when compared to control, *t* test. Images are representative blots of SIRT1 and GAPDH. M denotes molecular weight marker lane.

**Additional file 3: Figure S3.** Expression of SIRT1 in different regions of AD and control brain tissues. The levels of SIRT1 were determined in different regions of AD patients and were compared to a control-cohort. SIRT1 band intensity was normalised with GAPDH. Data are presented as fold change (±SD) with respect to control from three independent replicates with GAPDH used as an internal control housekeeping protein. **p < 0.01 and *p < 0.05 when compared to control, *t* test. Images are representative blots of SIRT1 and GAPDH. M denotes molecular weight marker lane.

## Abbreviations

SIRT1: sirtuin 1
SIR2: silent information regulator 2
NAD^+^: nicotinamide adenine dinucleotide
ROS: reactive oxygen species
PD: Parkinson’s disease
PDD: Parkinson’s disease with dementia
DLB: dementia with Lewy bodies
AD: Alzheimer’s disease
NF-κB: nuclear factor kappa-light-chain-enhancer of activated B cells
FOXO: forkhead box
PGC-1a: peroxisome proliferator-activated receptor gamma coactivator 1-alpha
PBS: phosphate buffered saline
DMSO: dimethyl sulfoxide
TBS: tris buffered saline
PEI: polyethyleneimine
NAM: nicotinamide
FCX: frontal cortex
TCX: temporal cortex
Cb: cerebellum
Pu: putamen
Hp: hippocampus
PMD: post-mortem delay

## References

[1] Tanno M (2007). Nucleocytoplasmic shuttling of the NAD^+^-dependent histone deacetylase SIRT1. J Biol Chem.

[2] Guarente L (2013). Calorie restriction and sirtuins revisited. Genes Dev.

[3] Lin SJ, Defossez PA, Guarente L (2000). Requirement of NAD and SIR2 for life-span extension by calorie restriction in *Saccharomyces cerevisiae*. Science.

[4] Satoh A (2013). Sirt1 extends life span and delays aging in mice through the regulation of Nk2 homeobox 1 in the DMH and LH. Cell Metab.

[5] Mercken EM (2014). SIRT1 but not its increased expression is essential for lifespan extension in caloric-restricted mice. Aging Cell.

[6] Mitchell SJ (2014). The SIRT1 activator SRT1720 extends lifespan and improves health of mice fed a standard diet. Cell Rep.

[7] de Lau LM, Breteler MM (2006). Epidemiology of Parkinson’s disease. Lancet Neurol.

[8] Tanner CM (1992). Epidemiology of Parkinson’s disease. Neurol Clin.

[9] Beitz JM (2014). Parkinson’s disease: a review. Front Biosci (Schol Ed).

[10] Hori YS (2013). Regulation of FOXOs and p53 by SIRT1 modulators under oxidative stress. PLoS ONE.

[11] Pallas M (2008). Modulation of SIRT1 expression in different neurodegenerative models and human pathologies. Neuroscience.

[12] Mudo G (2012). Transgenic expression and activation of PGC-1α protect dopaminergic neurons in the MPTP mouse model of Parkinson’s disease. Cell Mol Life Sci.

[13] Nisar R (2015). Diquat causes caspase-independent cell death in SH-SY5Y cells by production of ROS independently of mitochondria. Arch Toxicol.

[14] Brunet A (2004). Stress-dependent regulation of FOXO transcription factors by the SIRT1 deacetylase. Science.

[15] Campeau E (2009). A versatile viral system for expression and depletion of proteins in mammalian cells. PLoS ONE.

[16] Bradford MM (1976). A rapid and sensitive method for the quantitation of microgram quantities of protein utilizing the principle of protein-dye binding. Anal Biochem.

[17] Di Monte DA, Lavasani M, Manning-Bog AB (2002). Environmental factors in Parkinson’s disease. Neurotoxicology.

[18] Aquilano K (2010). Peroxisome proliferator-activated receptor gamma co-activator 1α (PGC-1α) and sirtuin 1 (SIRT1) reside in mitochondria: possible direct function in mitochondrial biogenesis. J Biol Chem.

[19] Poulose N, Raju R (2015). Sirtuin regulation in aging and injury. Biochim Biophys Acta.

[20] Jin Q (2007). Cytoplasm-localized SIRT1 enhances apoptosis. J Cell Physiol.

[21] Oberdoerffer P (2008). SIRT1 redistribution on chromatin promotes genomic stability but alters gene expression during aging. Cell.

[22] Lynch CJ (2010). SIRT1 undergoes alternative splicing in a novel auto-regulatory loop with p53. PLoS ONE.

[23] Giasson BI (2000). Oxidative damage linked to neurodegeneration by selective α-synuclein nitration in synucleinopathy lesions. Science.

[24] Feng Y (2015). Rotenone affects p53 transcriptional activity and apoptosis via targeting SIRT1 and H3K9 acetylation in SH-SY5Y cells. J Neurochem.

[25] Pfister JA (2008). Opposing effects of sirtuins on neuronal survival: SIRT1-mediated neuroprotection is independent of its deacetylase activity. PLoS ONE.

[26] Ghosh HS (2007). Sirt1 interacts with transducin-like enhancer of split-1 to inhibit nuclear factor kappaB-mediated transcription. Biochem J.

[27] Vallabhapurapu S, Karin M (2009). Regulation and function of NF-κB transcription factors in the immune system. Annu Rev Immunol.

[28] Morgan MJ, Liu ZG (2011). Crosstalk of reactive oxygen species and NF-κB signaling. Cell Res.

[29] Schmitz ML (2004). NF-κB: a multifaceted transcription factor regulated at several levels. ChemBioChem.

[30] Ryan KM (2000). Role of NF-κB in p53-mediated programmed cell death. Nature.

[31] Chaitanya GV, Steven AJ, Babu PP (2010). PARP-1 cleavage fragments: signatures of cell-death proteases in neurodegeneration. Cell Commun Signal.

[32] Rajamohan SB (2009). SIRT1 promotes cell survival under stress by deacetylation-dependent deactivation of poly(ADP-ribose) polymerase 1. Mol Cell Biol.

[33] Spillantini MG (1998). α-Synuclein in filamentous inclusions of Lewy bodies from Parkinson’s disease and dementia with lewy bodies. Proc Natl Acad Sci USA.

[34] Kim GH (2015). The role of oxidative stress in neurodegenerative diseases. Exp Neurobiol.

[35] Li S (2016). Cortical and subcortical changes in Alzheimer’s disease: a longitudinal and quantitative MRI study. Curr Alzheimer Res.

[36] Gandhi S, Abramov AY (2012). Mechanism of oxidative stress in neurodegeneration. Oxid Med Cell Longev.

[37] Glass CK (2010). Mechanisms underlying inflammation in neurodegeneration. Cell.

