# Supplementary figures and images for: SIRT1 ameliorates oxidative stress induced neural cell death and is down-regulated in Parkinson’s disease
Source: BMC Neurosci. 2017 Jun 2;18:46. doi: 10.1186/s12868-017-0364-1 (PMC5455114; doi:10.1186/s12868-017-0364-1)

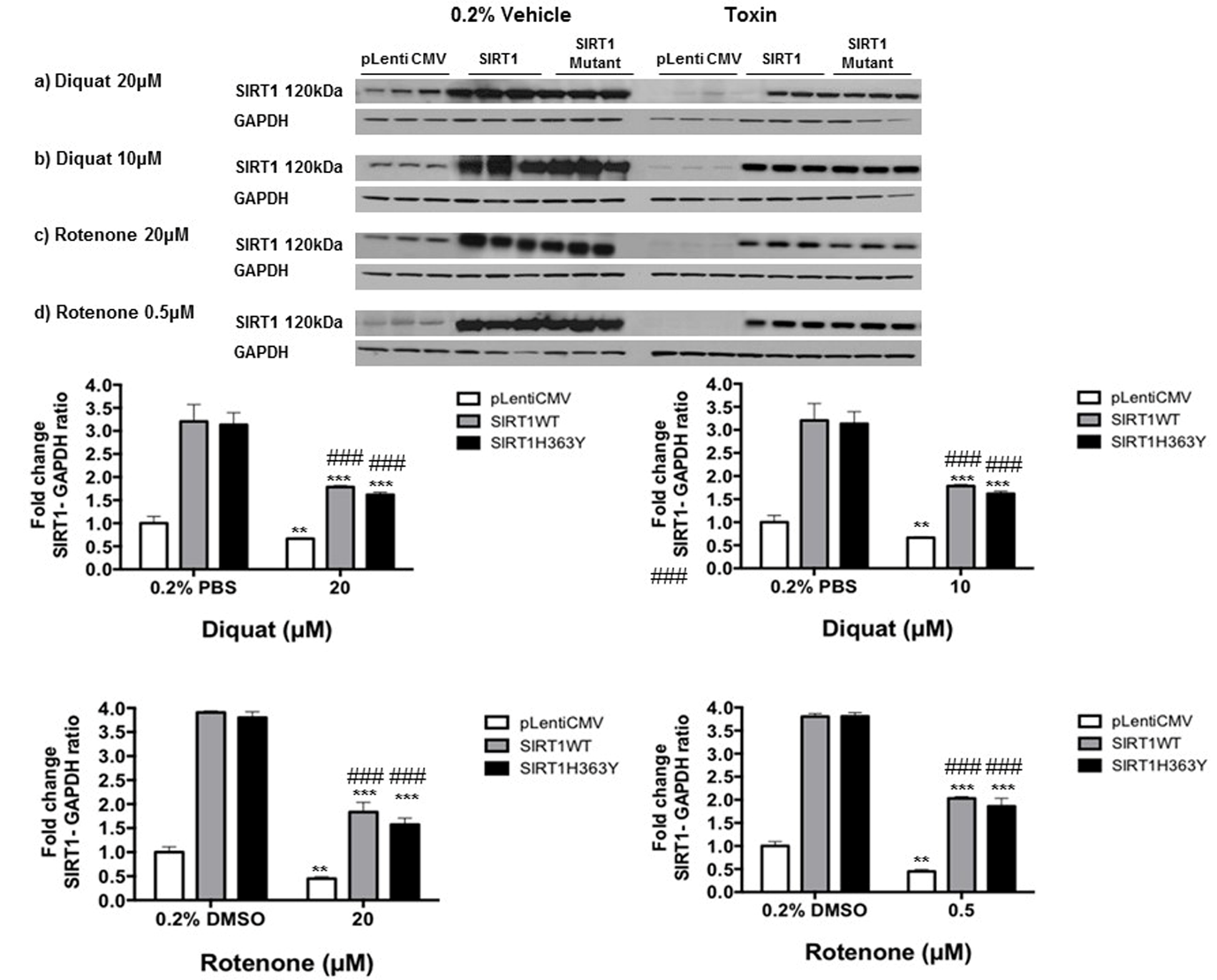


Additional file 1: Figure S1.

Supplement: Supplementary file 1 — Additional file 1: Figure S1. Expression of SIRT1 in toxin treated SH-SY5Y cells. SIRT1WT and SIRT1H363Y were over-expressed in SH-SY5Y cells and control cells were transfected with empty vector following which cells were treated with diquat (20 or 10 μM) or rotenone (20 or 0.5 μM) for 20 h. Cells were harvested and the samples were probed for SIRT1. Data are presented as fold- untreated (+SD) from three independent assays (n = 3) with comparison to GAPDH as a housekeeping control protein. ***p < 0.001 when compared to 0.2% PBS, one-way ANOVA (Bonferroni corrected), ###p < 0.001 when compared to empty vector treatment, two-way ANOVA (Bonferroni corrected). Images are representative blot of SIRT1 and GAPDH. [file 12868_2017_364_MOESM1_ESM.docx]

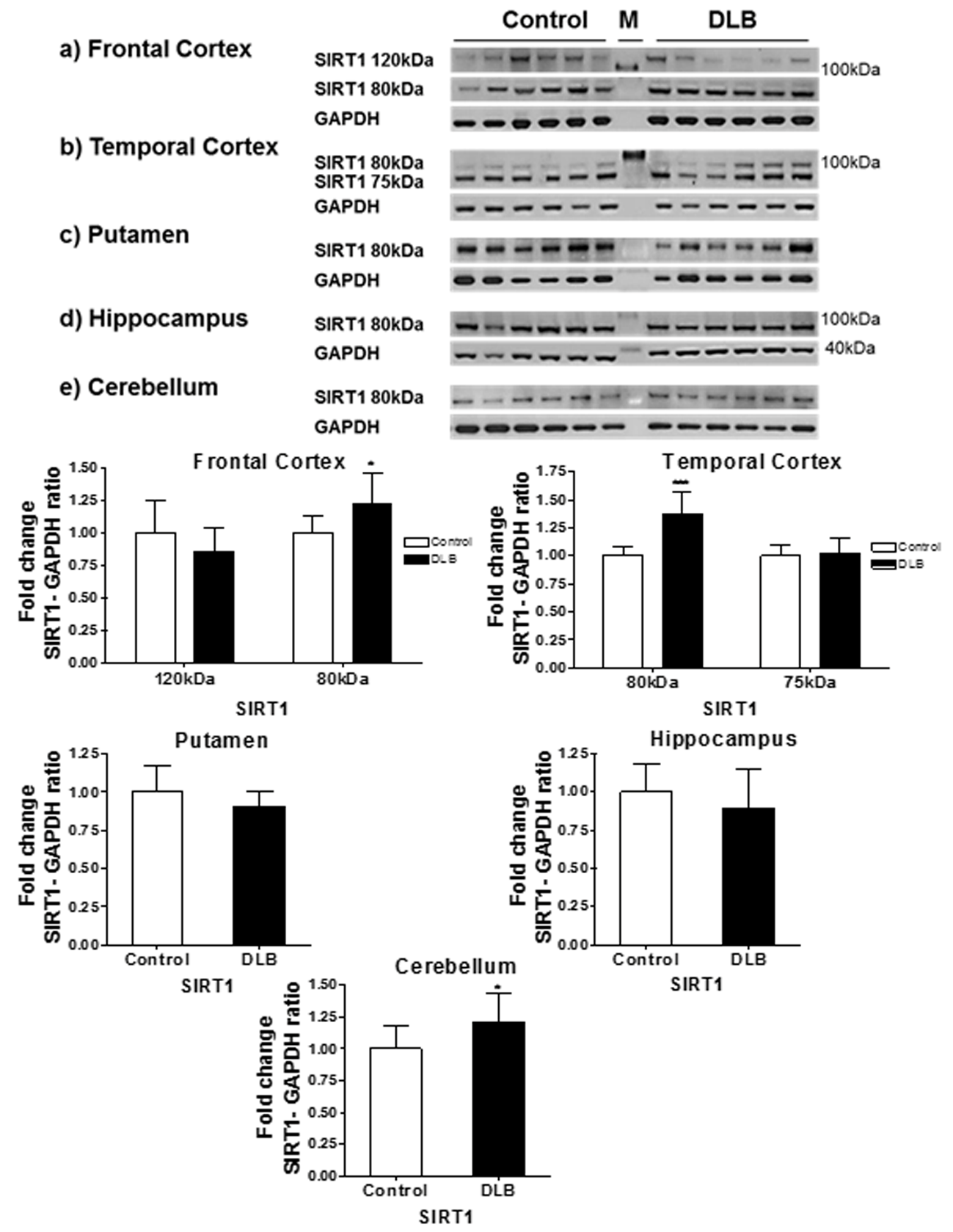


Additional file 2: Figure S2.

Supplement: Supplementary file 2 — Additional file 2: Figure S2. Expression of SIRT1 in different regions of DLB and control brain tissue. The levels of SIRT1 were determined in different regions of DLB patients and were compared to a control-cohort. SIRT1 band intensity was normalised with GAPDH. Data are presented as fold change (±SD) with respect to control from three independent replicates with comparison to GAPDH as a housekeeping control protein. **p < 0.01 and *p < 0.05 when compared to control, t test. Images are representative blots of SIRT1 and GAPDH. M denotes molecular weight marker lane. [file 12868_2017_364_MOESM2_ESM.docx]

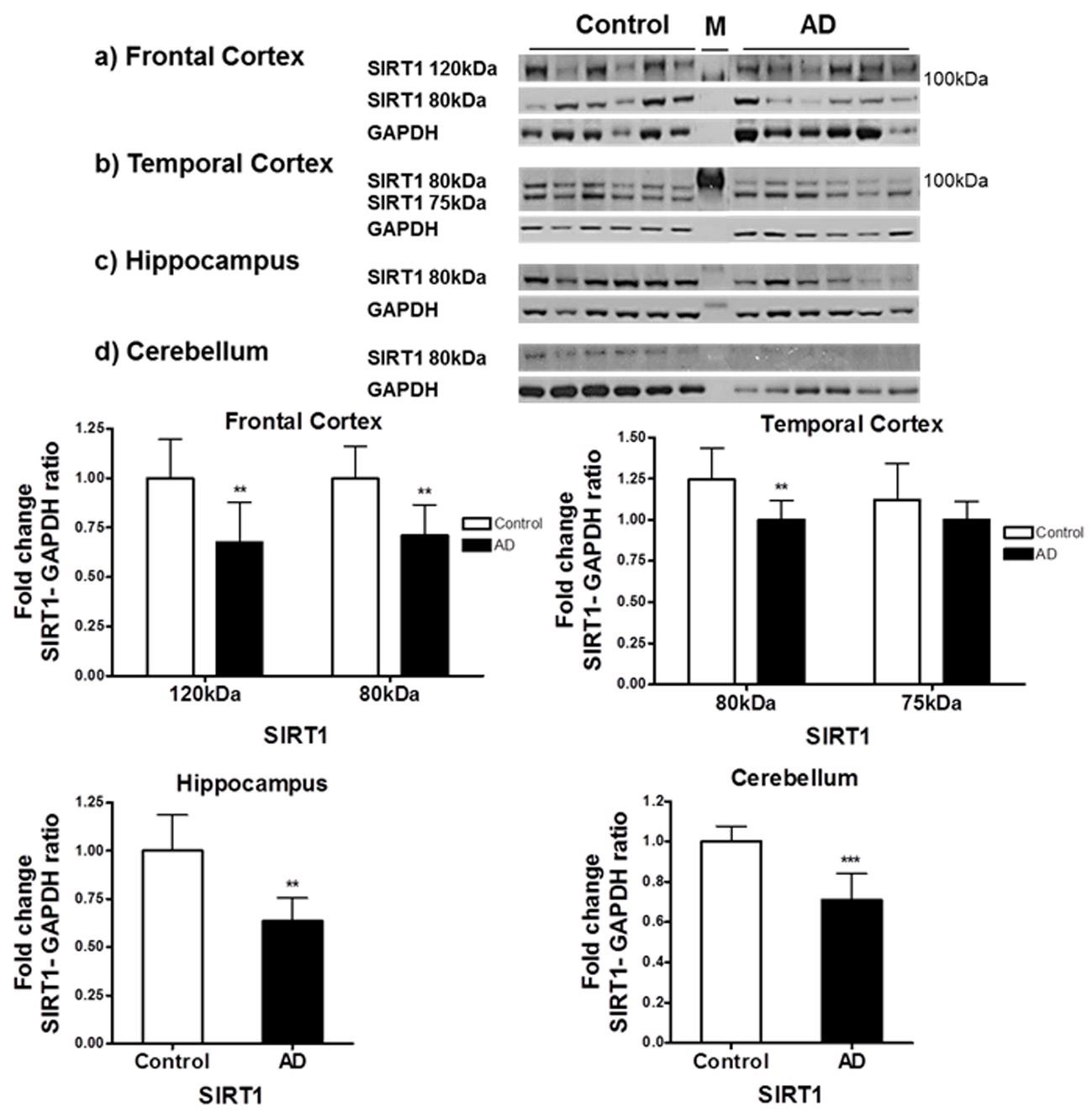


Additional file 3: Figure S3.

Supplement: Supplementary file 3 — Additional file 3: Figure S3. Expression of SIRT1 in different regions of AD and control brain tissues. The levels of SIRT1 were determined in different regions of AD patients and were compared to a control-cohort. SIRT1 band intensity was normalised with GAPDH. Data are presented as fold change (±SD) with respect to control from three independent replicates with GAPDH used as an internal control housekeeping protein. **p < 0.01 and *p < 0.05 when compared to control, t test. Images are representative blots of SIRT1 and GAPDH. M denotes molecular weight marker lane. [file 12868_2017_364_MOESM3_ESM.docx]
